# Older Adults Using *Our Voice* Citizen Science to Create Change in Their Neighborhood Environment

**DOI:** 10.3390/ijerph15122685

**Published:** 2018-11-28

**Authors:** Anthony G. Tuckett, Abbey Freeman, Sharon Hetherington, Paul A. Gardiner, Abby C. King, On behalf of Burnie Brae Citizen Scientists

**Affiliations:** 1 Director, Postgraduate Coursework Programs (Nursing, Midwifery), The University of Queensland, St Lucia, QLD 4072, Australia; 2 School of Nursing, Midwifery and Social Work, The University of Queensland, St Lucia, QLD 4072, Australia; a.freeman@uq.net.au; 3 Healthy Connections Exercise Clinic, Burnie Brae Ltd., Chermside, QLD 4032, Australia; Hetherington.s@burniebrae.org.au; 4 Centre for Health Services Research, Faculty of Medicine, The University of Queensland, St Lucia, QLD 4102, Australia; p.gardiner@uq.edu.au; 5 Department of Health Research and Policy and Medicine, Stanford Prevention, Research Center, Stanford University School of Medicine, Palo Alto, CA 94305, USA; king@stanford.edu

**Keywords:** older adult, physical activity, social connectedness, physical environment, citizen science, Discovery Tool

## Abstract

Physical activity, primarily comprised of walking in older adults, confers benefits for psychological health and mental well-being, functional status outcomes and social outcomes. In many communities, however, access to physical activity opportunities are limited, especially for older adults. This exploratory study engaged a small sample (*N* = 8) of adults aged 65 or older as citizen scientists to assess and then work to improve their communities. Using a uniquely designed mobile application (the Stanford Healthy Neighborhood Discovery Tool), participants recorded a total of 83 geocoded photos and audio narratives of physical environment features that served to help or hinder physical activity in and around their community center. In a facilitated process the citizen scientists then discussed, coded and synthesized their data. The citizen scientists then leveraged their findings to advocate with local decision-makers for specific community improvements to promote physical activity. These changes focused on: parks/playgrounds, footpaths, and traffic related safety/parking. Project results suggest that the *Our Voice* approach can be an effective strategy for the global goals of advancing rights and increasing self-determination among older adults.

## 1. Introduction

### 1.1. Population Ageing

In 2030 there will be 1 billion older adults globally (12% of the total population) [1]. Across the planet, the number of older adults is growing faster than the number of people in any other age group [2]. We can expect a 150% expansion of the population aged 65 and over in the next 35 years [1]. Worldwide the population 80 and over is projected to more than triple between 2015 and 2050 from 126.5 million to 446.6 million [1]. The WHO notes that reducing severe disability from disease and health conditions within this age cohort is one key to constraining health and social costs [3]. It is recommended that we need specifically targeted policies to address the needs of older adults to include housing, employment, health care, social protection and other forms of intergenerational support [2].

### 1.2. Physical Activity

For nearly all older adults, declining muscle mass, muscle strength and physical performance and increasing time spent sedentary are common pathways to disease, disability, falls risk, dependency and morbidity [4,5,6,7]. Consequent to this are the attendant care needs, community care costs and rising residential aged care uptake.

The evidence of the benefits of physical activity for the prevention and management of chronic conditions for older adults is well documented [6,8,9]. Pertinent to this study is the evidence that physical activity, primarily comprised of walking in older adults, confers benefits for psychological health and mental well-being (e.g., anxiety, depression, life satisfaction), functional status outcomes (e.g., sarcopenia, physical and cognitive function), and social outcomes (e.g., community involvement and social networks) [1,5,10,11]. Local environments can in turn affect physical activity levels. In addition to the effects of social aspects of the environment on physical activity, an extensive amount of evidence underscores the importance of physical environment features (e.g., intersection crossings, footpaths) on physical activity levels in different age groups.

### 1.3. Social Connectedness

Enhancing social participation is a central component of the WHO’s response to concerns about population ageing [12]. In its many forms social connectedness (social networks, social integration, social embeddedness, human companionship) is vital for active ageing [13]. Social ties help humans build resilience in the face of various hardships and help to extend our lives. They can matter even more as we age when ill health increases, which can diminish opportunities for social engagement [14]. Those who have larger networks tend to have better health especially when the interaction with network members is frequent [15]. Generally speaking, adults who are more socially connected are healthier and live longer than their isolated peers; there is also a direct relationship between poor mental health and risk of dying which can be influenced by low quantity and quality of social relationships [16]. Social ties influence health behaviors and physical health [16].

Social connectedness and engagement can be affected by the surrounding physical environment in which older adults live. For example, proximity to parks and community centers can influence the amount of social engagement as well as physical activity that older adults obtain on a regular basis. Local community senior centers and volunteer organizations can play an important role in facilitating engagement with social activities. Social groups occurring in neighborhoods or at nearby community venues are typically ‘cheap to run’ (they do not require a trained other to manage them) and because of their contribution to better bio-psychological health and health behaviors across time, their capacity to build social connectedness can contribute to lower healthcare costs and greater quality of life [11,16].

### 1.4. Physical Environment

The associations between neighborhoods and health have been reported to be the strongest among adults around retirement age [17]. Furthermore, the positive impacts of walking on older adults’ strength and flexibility supports the value of walking in warding off disability and extending older adults’ capacity for independence and aging in place [18]. Planners and civil engineers are therefore groups that can contribute to ‘…developing new and redeveloping existing communities to address the health, safety and mobility of older adults’ [18] (p. 43). This latter sentiment is supported elsewhere: ‘policy makers should consider how to provide both psychosocial and physical environment resources to support seniors’ physical activity’ [19] (p. 73). Hence, urban planners have been increasingly encouraged to incorporate into their designs walkable neighborhoods which by definition are characterized by mixed land use, interconnecting streets, convenient transit locations and compact communities [20]. What is currently less clear are the types of neighborhood features that would be particularly helpful for promoting regular walking among older adults across the continuum of mobility as people age. A valid and reliable approach for older adults to inform urban planners and policy-makers about older adults’ needs related to their physical environment is through citizen science.

### 1.5. Citizen Science “by the People”

**Figure 1:**
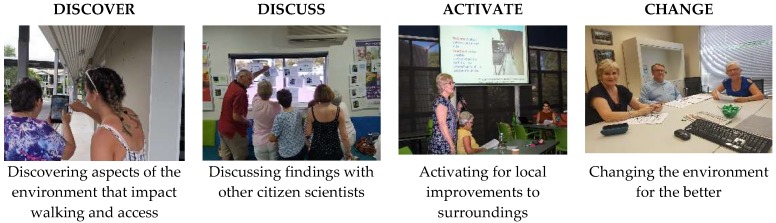
Our Voice Citizen Scientist framework (King et al., 2016) [21]. © Stanford University. All rights reserved.

The *Our Voice* Citizen Science model is a community-based empowerment approach in which citizen scientists are trained and supported to become agents of change in their own communities [21]. In the “Discover” phase of this approach, citizen scientists use a simple mobile application called the Stanford Healthy Neighbourhood Discovery Tool (DT), to document local environmental features through geo-coded photographs, audio narratives, and walking routes. The DT is user-friendly across all levels of education and technology literacy, and has been operated successfully by persons ages 10 to 92. In the facilitated “Discuss” phase, citizen scientists review and analyze their own data in order to collectively identify and prioritize challenges. The group then moves into the “Activate” phase, brainstorming potential solutions and working with local community partners to develop and advocate for realistic, low-cost changes to local structures, policies and practices (see Figure 1). This process gives participants a “voice”—a means of telling their stories, building consensus, and motivating action. *Our Voice* blends the active participant engagement and ownership inherent in community-based participatory research with the standardized participant-based data collection methods that are a hallmark of citizen science. This approach allows citizen scientists to merge individual storytelling to represent collective experiences and advocate for changes that will impact both individuals and their communities.

Research has demonstrated that this approach works. Having older adults evaluate their neighborhoods and then meet with local and public sector decision makers for the purpose of bringing improvements to these neighborhoods is a viable approach [22]. Citizen science empowers older adults to participate more actively in local management decisions [23] and policy-making [23]. Accordingly, ‘engaging older adult community residents in advocacy has the potential to achieve better local policy outcomes, improve […] physical activity environments, and provide direct benefit to the residents’ physical health and well-being’ [24]. As Miller [25] suggests, community development works best when social change is initiated from bottom-up support and actions. The approach lends itself to improved knowledge transfer impacting policy and action [26,27]. Specific to research with older adults as citizen scientists, Buman and his colleagues concluded that community-focused values and interests bought by the older adults themselves guarantees that policy decisions reflect the older adults’ priorities and beliefs [24]. The purpose of this study was to apply the *Our Voice* citizen science model with older adults affiliated with the Burnie Brae community center, engaging and empowering those participants to document their lived experiences and drive positive changes in their local environment.

#### Research questions

1. What are the features that help or hinder access to a seniors’ center?
2. What are the features of the physical environment surrounding a seniors’ center that help or hinder physical activity (walking)?
3. In what ways can older adults acting as citizen scientists bring about changes to their local environment?

## 2. Methods

This study applied the *Our Voice* citizen science framework (Figure 1) [27]. *Our Voice* is a community partnership process aimed at better understanding community members’ lived experiences—supporting outreach and action as a direct consequence of a collaborative research project [28].

Burnie Brae (the Chermside and District Senior Citizens Centre Incorporated, Queensland, Australia) is a large not-for-profit and charitable organization established in Brisbane (Australia) in 1984. It is Queensland’s largest over 50’s community center. Burnie Brae operates across four locations on the north side of Brisbane (Australia) and offers a wide range of activities to its membership base of more than 6000 (average age 69 years). The Burnie Brae Centre is adjacent to Burnie Brae Parklands—an area of approximately 3.91 hectares (0.0391 km^2^). Based on an ongoing academic partnership, the Centre Executive invited the research to be undertaken.

Data were collected from January to March 2018. All citizen scientists gave their informed consent for inclusion before they participated in the study. The study was conducted in accordance with the Declaration of Helsinki, and the protocol was approved by the University of Queensland Human Research Ethic Committee (Approval number #2017000913) as well as approval through the Burnie Brae Research Administration Group (September 2017).

### 2.1. Participants and Recruitment

#### 2.1.1. Citizen Scientists

To be eligible to participate in this study, citizen scientists had to be members of the Burnie Brae, aged 65 years or older, English speaking, actively engaged in a range of social activities (e.g., volunteer gardening, attended the gymnasium and/or participating in organized social outings) and able to walk unaided (i.e., independent of walking aids of any kind). Participants were recruited from December 2017–January 2018 via posters displayed at Burnie Brae with further person-to-person invitations through the Burnie Brae subsidiary Healthy Connections Exercise Clinic. People registered their interest and attended a 60-min group face-to-face information session. At this information session they were given a detailed verbal explanation of the study (using a Power Point presentation) that included a YouTube © video (www.youtube.com/watch?v=sYcYXh51Bl0), safety instructions and instructions not to photograph persons’ faces/identifiable features. Attendees were assessed for eligibility based on the inclusion/exclusion criteria with all found eligible. Citizen scientists were also provided a hard copy project information sheet and provided written informed consent and they also signed a photography release form.

#### 2.1.2. Researchers (Partner Facilitators)

Eight citizen scientists partnered with members of the research team and a research assistant in the implementation and emerging design of this research. All but one of the citizen scientists was female, and all were independently mobile and 65 years or over. Researchers AT and PG are active members of the Stanford University Citizen Science Global Research Network (CSGRN see: http://med.stanford.edu/ourvoice.html), AF is a senior undergraduate dual-degree nursing/ midwifery scholar who undertook a short summer research internship and SH is a Burnie Brae researcher and Accredited Exercise Physiologist (AEP).

#### 2.1.3. Enabling Actors (Decision Makers)

Burnie Brae Executives and the Member for Northgate-Brisbane City Council, were the purposefully targeted decision-makers. For Burnie Brae this included the Chief Executive Officer (CEO), the Chairman of the Board (COB) and the Operating Manager (OM). Municipal Councillor AA is the elected representative for the Brisbane City Council and has local jurisdiction over a Council Parks Team, the Transport Planning and Strategy Branch, and the Field Services Team responsible for repair and improvements to surrounding parklands, footpaths and roadways in the immediate neighborhood. The CEO, COB and OM were invited by AT to hear the findings and the CEO later also met with AT and the citizen science advocates (HP, NK) to reach an agreement on actions. Councillor AA was invited through the Chairman of the Board to attend a further hearing of findings and proposed solutions in the company of the citizen scientist pair (DW, CL) and Chairman of the Board and co-researchers AT and PG.

#### 2.1.4. Discovery: Data Collection by Citizen Scientists

To discover aspects of the local neighborhood environment, the citizen scientists walked around the Burnie Brae center and the adjacent park. During these walks, the citizen scientists used the Stanford Healthy Neighborhood Discovery Tool (Version 1.9.6) (Stanford University, Palo Alto, CA, USA) application on a Nexus tablet. The Stanford Healthy Neighborhood Discovery Tool (DT) is a non-medical mobile application created by Stanford University. It is licensed for use by contributing research projects at outside institutions for the purpose of data collection only. The DT is designed for single use by community members to gather environmental photos (explicitly not to include identifiable persons), audio narratives, and GPS-tracked walking routes. Users also answer a brief 8-question survey that captures basic non-identifiable demographic information and assesses perceptions of the neighborhood environment [29]. This app is designed to record physical environment (neighborhood features) and building access points that citizen scientists perceived impacted walking or accessing their center [29]. Citizen scientists individually designated a feature as helpful or a hindrance on the DT app. by clicking on a green smiley face or red angry face after reviewing each image. Citizen scientists were accompanied by a trained member of the research team who provided verbal instructions on how to use the app. The accompanying trained member of the research team did not influence the data recorded but served as a silent observer to ensure the safety of the participant [30] and, where required, to provide technical support [31]. The neighborhood walk canvassed the outside building facilities and amenities, parking areas and surrounding Burnie Brae Park (http://www.brisparks.com.au/qld/chermside/burnie-brae-park). Four participants undertook the discovery phase of the study immediately following the information session, with the remaining four participants completing the walk within 3 days (*N* = 3) and 15 days (*N* = 1) of the information session, respectively (see Figure 1).

##### Data Storage and Security

Data are uploaded to a password protected Discovery Tool Data Repository (DTDR) and stored on a secure server at the Stanford University School of Medicine. All data uploaded to the password-protected data repository are backed up with remote data storage servers. The servers are maintained by Stanford IT managers. Access to these data are managed by a Protocol Director. The data are kept securely within the Stanford University system for up to 30 years. Anonymous data are collected with the DT. No personal health information is recorded or stored. Data uploaded to the DTDR are not linked to individual participants. Any identifiable photos are immediately blurred or removed from the server. The audio narratives recorded on the DT are the only potentially-identifiable data that is stored in the DTDR. Project data collected using the DT and stored in the DTDR may be shared via a password-protected website only with those collaborators who have signed the DT License Agreement and submitted and complied with an approved study protocol; and human subjects/ethics standards from their home institution.

### 2.2. Data Preparation by Research Team Members

All attendant geocoded narratives were transcribed and photographs downloaded and printed in high resolution color. These were then placed in named plastic sleeves corresponding to the citizen scientists (A–H). The day after the final walk (late January 2018) two members of the research team (AT, AF) met to prepare the data for the first community meeting where the data were discussed (see Figure 1). The research team relied on the initial 19 content elements derived from a previous developed coding schema [29,30]. It was deemed necessary to replace some code element terminology (for culturally specific reasons); to merge some code elements (to be specific to the neighborhood walk for this precinct) and remove some altogether (where no data were obviously forthcoming) (see Table 1).

### 2.3. Discuss: Data Analysis by Citizen Scientists

After completion of the last walk and data preparation, the discuss phase was enacted and citizen scientists gathered to code their own data (see Figure 1). Co-researcher AT reiterated and further explained the process and facilitated the citizen scientist group.

Code element headings based on the validated coding schema comprising our revised 10 elements [29,30] were affixed to the walls of the meeting place. The citizen scientists assigned their photographs, paired with transcribed audio narratives, on the wall under the appropriate heading. In addition, the citizen scientists allocated positive data on one side of each element space and negative data on the other. The benefit of this was that it created an immediate visual impression of what the most frequently noted content element and hindrances were. Once each citizen scientist had completed their data assignment, the group walked around to each of the 10 content elements for the purpose of reaching consensus. The data from one citizen scientist, who was unable to attend this session, were not analyzed.

At this point the group nominated two citizen scientist advocates to be the group’s representatives to take the findings and their solutions to the activate session (see Figure 1). One of the citizen science advocates (HP) led the discussion to prioritize three hindrances to walking and/or center access that they wanted addressed. Determinations by the citizen scientists of the most important barriers in their physical environment that hinder physical activity were facilitated by them tallying the negative data assignment (see Table 2). Finally, a collective brainstorming of solutions took place. Priorities and solutions were written on a whiteboard and recorded photographically and by the researcher taking notes of the session.

### 2.4. Activate: Voicing the(ir) Needs and Prioritising Solutions by Citizen Scientists

Two activate sessions were driven by the citizen scientists-activate session one was a public presentation open to the center membership and the eight citizen scientist co-researchers (see Figure 1); activate session two was a ‘closed’ citizen science presentation to the municipal council member. Activate session one took place in the first week of February and activate session two took place in third week of March, 2018.

At activate session one, the nominated citizen science advocates delivered a 55-minute, Power-Point presentation followed by a question and answer session. This session focused on the group’s needs (*N* = 7), and their proposed solutions to improve the features of the physical environment surrounding their center to encourage physical activity (walking). The audience comprised the enabling actors, five citizen scientists and three members of the research team, and the research assistant. The citizen science advocates had input into the design of the Power-Point presentation; were provided a presentation script (based on the co-designed Power-Point presentation) in the days before the session and arrived on the day one hour before the session to practice their oral presentation and Power-Point skills with AF and AT. A week following this activate session one, the CEO of Burnie Brae met again with the two citizen science advocates and co-researcher AT to further discuss and commit to changing the physical environment features that needed improvement and over which the CEO had jurisdiction.

At activate session two, the nominated citizen science advocates were unavailable to attend due to prior commitments. Instead, citizen scientists DW and CL advocated for the group. With the change to the citizen scientists acting as the groups’ advocates, the Power- Point presentation and question and answer session was co-delivered with AT. Present at this session were the municipal council member AA, Burnie Brae Chairman of the Board and co-researcher PG. The focus of the session was also the physical environment features that needed improvement and over which the Councillor had jurisdiction and thus responsibility through the municipal council. It was here that the Councillor committed to making the citizen scientists’ proposed changes.

### 2.5. Change: Citizen Scientists Making a Real Difference

At the time of writing, the planning continues for the work related to Burnie Brae Centre access and the commitments to physical environment features that needed improvement, and over which Burnie Brae’s CEO has jurisdiction. Councillor AA, as the elected representative for the Brisbane City Council, has instructed his Council Parks Team, the Transport Planning and Strategy Branch, and the Field Services Team responsible for repair and improvements to surrounding parklands, footpaths and roadways to commence work. Footpath repairs and have started. Line marking road works are completed. A derived outcome from this project has been the approval for the construction of a new toilet block and additional exercise equipment in the Park.

## 3. Results: Citizen Scientist Participation and Data Collected

Eight citizen scientists participated in this study. Most were women (87.5%, 7/8), and all were able to walk independently. All were 65 years or over. 87.5% (7/8) rated the app ‘extremely useful’ with the one other rating the app. ‘somewhat useful’. All but one of the citizen scientists was contacted both by telephone and attended the information session. Seven attended the discuss session with five attending activate session 1 and two activate session 2 (see Figure 1).

The citizen scientists took a total of 83 photographs and recorded 83 audio commentaries of the photographs (9–11 photos/commentaries per citizen scientist) on an average walk duration of 18 min. Table 2 shows the number of images by coded elements from the discuss session. The three issues prioritized by the citizen scientists to advocate for were parks/playgrounds, footpaths, and traffic related safety/parking.

The following represents examples of the citizen scientists’ findings and solutions under the three priority issues (features of the physical environment surrounding a senior’s centre that hinder physical activity). Further audio-narratives that support these hindrances are provided in Table 3.

### 3.1. Parks/Playgrounds

**Figure 2:**
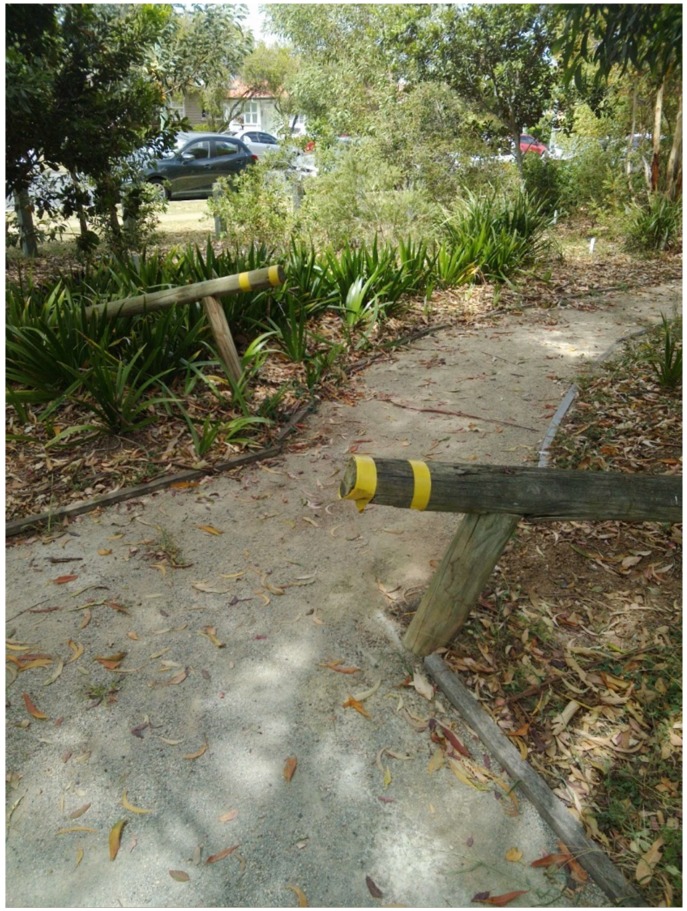
The sleeper.

Citizen scientist F and H recorded this environmental feature (Figure 2). Citizen scientist F narrated:

“I’m finding when you get to the end of the gravel path, there is a sleeper (a heavy timber beam, especially one that is laid horizontally on the ground), I’m not too sure if it’s supposed to be on a slope but one part is sticking out quite a bit that would cause some problem to people with walkers”

The feature was evaluated as a safety risk. During the discuss phase (see Figure 1) the citizen scientist group proposed that the nominated citizen scientist advocates investigate the purpose of this feature with Council, with a view to having it removed. When the advocate pair presented their finding at the community meeting and met with the Burnie Brae Chief Executive Officer, the issue was dealt with by Centre Grounds and Maintenance to make the hazard more visible as it could not be removed.

**Figure 3:**
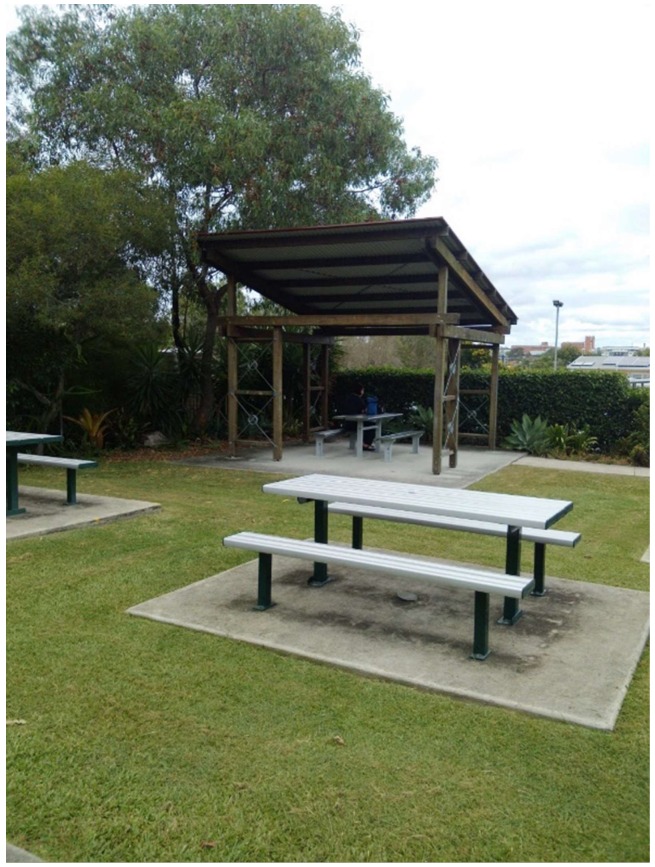
Shade.

Citizen scientist C recorded this feature (Figure 3) and citizen scientist F cautioned where these facilities do exist ‘you just have to be a little bit careful walking onto the cement slab’:

“More covered shade over the tables, there is three other tables, which would make it more convenient for a picnic and make it more pleasant”

Whilst other citizen scientists recorded favorably the shaded areas that do exist, the group felt that there remains a need to build more shaded shelter. This was a priority taken to the municipal Councillor.

**Figure 4:**
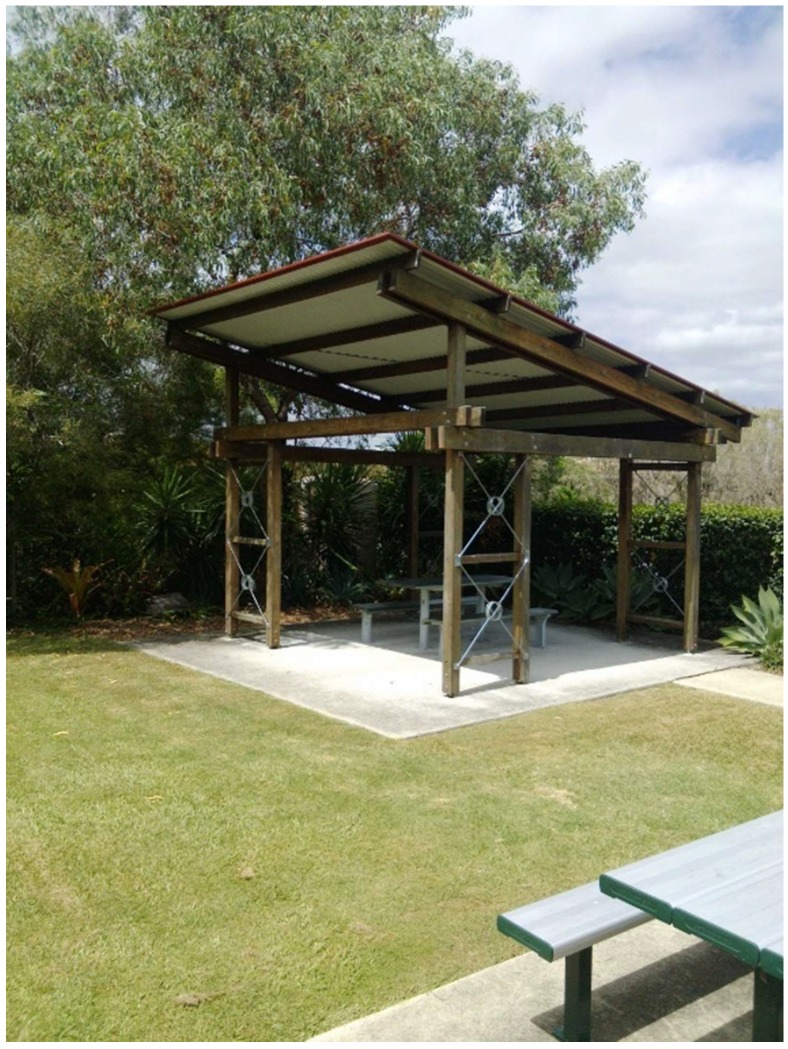
Urine stench.

Citizen scientist H noted the outside shade areas for a different reason (Figure 4), concerned about the impact on walking around Burnie Brae Park:

“This is a photo of urine stench, don’t know how it gets there or how to keep it away but it is very unpleasant”

It was recommended to the Burnie Brae Executive by the citizen scientist advocates that the group’s solution was to demand the municipal council to spray and clean the area regularly. However, the Executive group acknowledged the problem as a Centre issue and directed Grounds & Maintenance to clean and disinfect the area.

### 3.2. Footpaths

**Figure 5:**
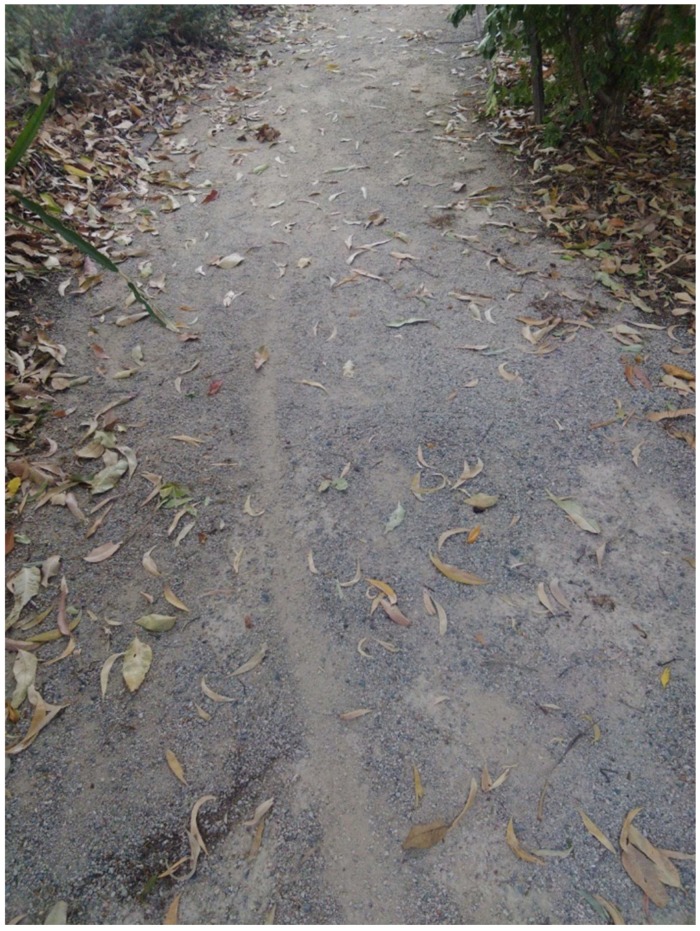
Loose gravel path.

Citizen scientist B recorded Figure 5. In contrast, it was the case that citizen scientists A and H agreed that the shared pathway (bike/pedestrian) in the park was a facilitator for their walking; and citizen scientist C recorded an impression of ‘safe pathways’. However, for citizen scientist B:

“Seems to be a loose gravel path, can’t quite see where it’s heading, but maybe it wouldn’t be a good idea for people with walkers”

The group supported this as an issue and proposed that signage be placed to alert persons that the path was a loose surface. At activate session one (1) the Executive group explained that the nature of the surface meant it was prone to scuffing. However, the CEO agreed that signage could be posted to alert persons that the path surface was loose (see Figure 1).

**Figure 6:**
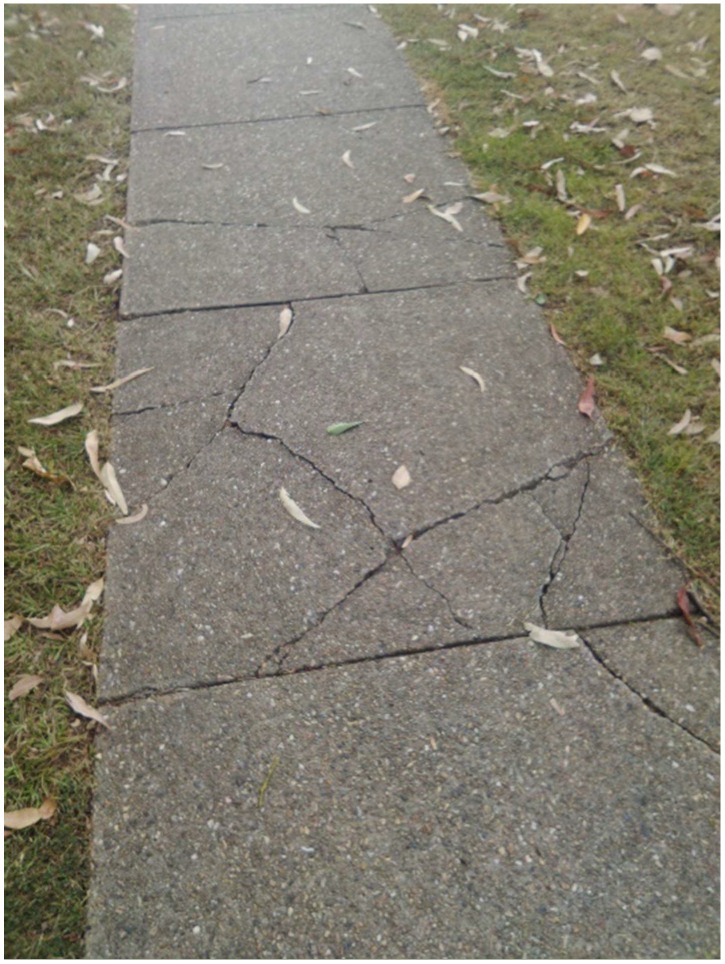
Damaged footpath.

Citizen scientist B recorded Figure 6 and was not alone (also citizen scientist H):

“Damaged footpath could be a hazard for people tripping”

This was a group consensus and citizen scientist advocates proposed a letter to be sent to the municipal council. In conversation with the CEO, it was further recommended that additional photographs be taken whereby the exact areas were precisely ‘spot-marked’ with paint. This data were then presented to the municipal Councillor. Preparatory repair work has started.

### 3.3. Traffic Related Safety/Parking

**Figure 7:**
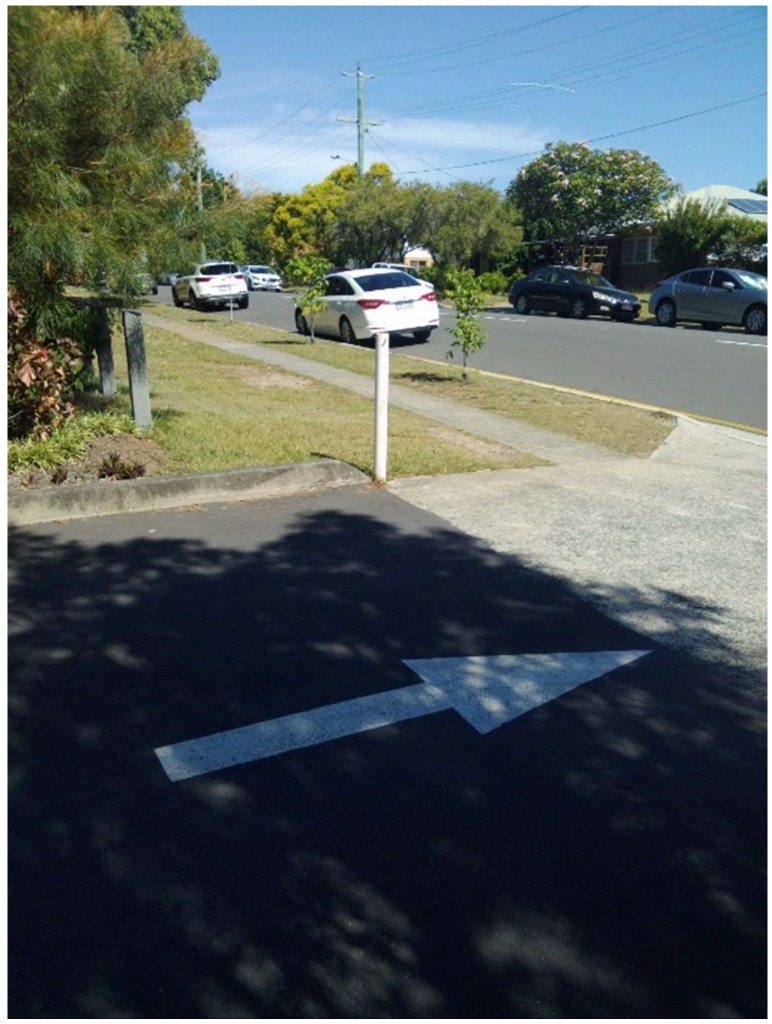
Likely to have an accident.

Citizen scientist A expressed real safety concern related to Figure 7. Given other recorded images and narratives about the lack of parking (‘car park always full’), the issue captured here takes on greater significance:

“On leaving the car park, it’s very difficult when you’re turning right with all the cars parked that you can’t see cars coming down on the left-hand side and you need to take it out very carefully or you’re likely to have an accident as all the cars on the left-hand side actually block your view”

The citizen scientists’ solution was to suggest the installation of a reverse mirror and/or to extend the yellow line road marking. This was a priority taken to municipal Councillor. The line marking has been completed.

**Figure 8:**
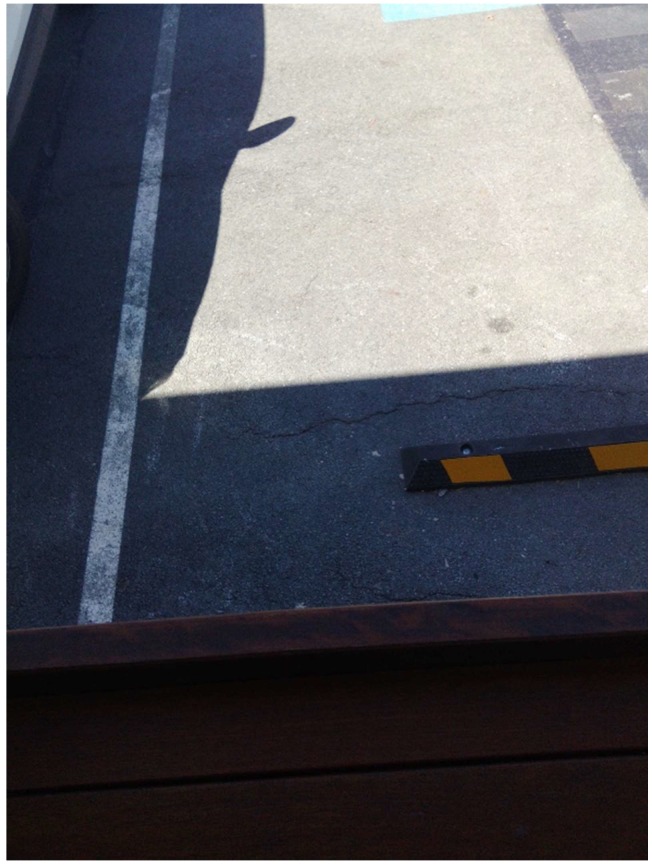
Carpark repainting.

Citizen scientist E recognized that safely walking about the outside of the center relied on there being suitable line markings in the car park (Figure 8).

“Carpark needs repainting for the lines in the carpark, it looks terrible”

The Centre CEO accepted the citizen scientists’ request that the line markings be redone.

Additional illustrative quotes for the hindrances to physical activity (walking) are included in Table 3.

## 4. Discussion

Our research allowed community dwelling older adults, as citizen scientists, to evaluate their physical environment around the Burnie Brae center for features that help or hinder their walking or access to it, respectively. These citizen scientists relied on smart technology in the form of the Discovery Tool to effectively capture digital geocoded images and record narratives about these features whilst undertaking a neighborhood walk. Collectively these citizen scientists analyzed their own data, allocating them to meaningful codes and from this they identified those hindrances to walking and access they wanted ameliorated. The group generated solutions which their nominated advocate pair presented to their center Chief Executive Officer, Chairman of the Board, Operating Manager; and the municipal Councillor at formal meetings. Adopting the *Our Voice* framework and the Discovery Tool for the first time in Australia, this study showed that it is possible for citizen scientists to enact changes in the neighborhood. 

We have suggested [32] that the nature of the research reported here extends original forms of photo voice research [33,34,35,36]. Here we add smart technology in the form of the Discovery Tool application to underscore the critical opinions of older adults who not only identify their needs and assets but also offer real-world, real-time solutions to their identified problem(s) [37].

The *Our Voice* framework has been successfully tested in a number of lower-income neighborhoods in California’s Bay Area (e.g., East Palo Alto, north San Mateo County, south Santa Clara County), and internationally in Mexico, Colombia, Chile and Israel [21]. These projects have addressed a range of challenges facing marginalized communities (e.g., walkability, food access, safe routes to school, park safety and land use) and have shown changes across multiple levels: individual, social, physical environment and policy [24,29,30,31,37]. An emerging Our Voice Citizen Science Global Research Network (CSGRN) (see http://med.stanford.edu/ourvoice.html) is advancing the paradigm in the USA-inspiring, for example, African-American woman to identify aspects of their local environments that if improved would support the daily habit of walking. In addition, the Our Voice CSGRN is targeting older adults (and adolescents) in Brazil, Chile, Manitoba (Canada), the UK and Taiwan as well as young adults and Maori communities in New Zealand.

We propose that there are a range of good reasons for the implementation of the *Our Voice* Citizen Scientist framework and community engagement process as an environmentally-focused intervention. Our proposals come with a caveat, namely the scope and reach of our findings must be set alongside our research limitations, which include a small sample size and reasonably short time frames in which impacts of the intervention have been evaluated. *Our Voice* projects are currently being conducted globally with larger numbers of citizen scientists (e.g., a hundred plus) and across longer time frames (e.g., two years).

The physical environment is a key to social participation which is vital to successful ageing [38,39]. The physical characteristics of the home, neighborhood and transport infrastructure all have a bearing on older adult’s ability to maintain independence and engage socially [40] The latter is protective of older adult’s physical, cognitive and mental health [41], and is facilitated through development of social capital (i.e., neighborhood trust, community belonging). The older adult as a citizen scientist, engaged in the way we have described, not only improves their immediate neighborhood to facilitate active living but also engages in a process that brings them together; sharing ideas, agreeing and disagreeing as they propose solutions that are meaningful to their immediate needs.

It is only by incorporating the perspectives and experiences of the citizens in our neighborhoods, communities and prefectures that we might expect to meet the UN 2030 Sustainable Development Goals of ‘making cities and human settlements inclusive, safe, resilient and sustainable’ [2]. Furthermore, the 2002 Madrid International Plan of Action on Ageing (MIPAA) recognizes that older adults ought to participate in and benefit equitably from the outcomes of development to advance their health and well-being and that societies should provide enabling environments for them to do so [42]. Increasingly, there is an expectation to create aged-friendly standards and environments to help prevent the onset or worsening of disabilities [42] and to design urban spaces free of barriers to mobility and access. Mindful of the earlier noted caveats, the citizen science we have described herein does all of these things.

Consistent with the 2002 MIPAA [42] our older adult as a citizen scientist is recognized as “participant(s) in development planning, emphasizing (they ought to be able to) participate in and benefit equitably from the (outcomes) of development to advance their health and well-being, and that societies should provide enabling environments for them to do so” [2] (p. 1). The participatory role of the public as citizen scientists in the *Our Voice* framework posits it as a perfect mechanism for decision making about the environment. *Our Voice* supports one of the core rights of the Aarhus Convention [43].

The WHO’s *Global Age-Friendly Cities Project* and guide contains some eight (8) core age-friendly features as universal standards for an age-friendly city [44]. The WHO project identified, by listening to the voices of older adults, two urban living areas of specificity for the research being reported here, namely: age-friendly outdoor spaces and buildings and age-friendly respect and social inclusion [44]. The former means that the older adult expects well-maintained and safe green spaces, pedestrian friendly walkways, well-maintained pavements and well-designed roads. The latter means that the older adult is consulted on ways to serve them better; and that the older adult is included as a ‘full partner in community decision-making affecting them’ [45] (p. 50). Across the international literature, an age-friendly city takes as a starting point older adult’s lives and experiences to identify desirable community services and support [46]. Mindful of our project’s small size, we propose that older adults as citizen scientists engaging in the *Our Voice* processes are participating in the very assessments the WHO suggests a city can make about its age-friendly urban features [44].

Scaled-up, *Our Voice* can be enacted to address these big topics espoused by the ‘big players’—we have named a few (e.g., UN 2030 Sustainable Development Goals; 2002 Madrid International Plan of Action on Ageing; UNECE Aarhus Convention). A planned future application for this work could also include persons with dementia (PWD), in the hope of driving change towards creating and maintaining dementia-friendly habitats [47].

### Limitations

This project utilized a small purposive sample of motivated members of the Burnie Brae Centre as citizen scientists. Outcomes need to be weighed carefully against sample size. Whilst the relatively small sample generated a range of issues in the physical environment around the Centre and the project achieved its stated aim of consolidating feedback and solutions from the member base on ways to improve the walkability of their environment, caution must be taken about extrapolating outcomes. Equally, the sample was taken from a single Centre and caution should be paid when translating findings to other locales. Lastly, the small sample was predominantly female. Though this is reflective of the Center membership, it can be reasonably assumed that features of the physical environment that are identified by older women might not necessarily be the same as features identified by older men.

## 5. Conclusions

Older adults are often the recipients of services and solutions that are provided to them by an external other; by government, institution or organization. In this paper, we present a project were issues have been identified and solutions have been formulated by the older adult for the older adult. Further, the older adults have actively advocated for the implementation of these solutions to the benefit of their entire community. As outlined in the discussion, this has the potential to satisfy on many levels the objectives set by world-leading organizations who demand rights for older adults and for them to be to self-determining in all aspects of their lives.

## Figures and Tables

**Table 1:** The coded elements used here to derive the three issues prioritized by the citizen scientists during the Discuss session, January 2018.

Code Element [29,30] (*N* = 19)	Code Element (*N* = 10)
Parks/playgrounds	Parks/playgrounds
Traffic Traffic related safety Parking	Traffic related safety/Parking
Sidewalks	Footpaths
Mobility/Access issues	Mobility/Access issues
Amenities/destinations	Amenities/destinations
Crosswalks	Zebra crossing
Other	Other
Street features	Street features
Aesthetics	Pretty surroundings
Trash	Rubbish/litter/filth
Private residences	
Crime/security	
Graffiti	
Other people	
Footbridge	
Vacant lots	
Dogs	

**Table 2:** The barriers and facilitators coded by citizen scientists at the Discuss session, ranked by total frequency, that hinder or help physical activity (walking) and access, January 2018.

Coded Elements	Total		Hinder		Help		Neutral	
	*N*	%	*N*	%	*N*	%	*N*	%
Parks/playgrounds	24	33	9	13	14	19	1	1
Footpaths	12	17	6	8	5	7	1	1
Pretty surroundings	11	15	0	0	11	15	0	0
Traffic related safety/Parking	8	11	7	10	1	1	0	0
Mobility/Access issues	8	11	4	6	4	6	0	0
Other	5	7	1	1	3	4	1	1
Amenities/destinations	3	4	3	4	0	0	0	0
Zebra crossings	1	1	1	1	0	0	0	0
Street features	0	0	0	0	0	0	0	0
Rubbish/litter/filth	0	0	0	0	0	0	0	0

**Table 3:** Examples of audio narratives (hindrance) to physical activity (walking) and access made by citizen scientists using the Discovery Tool during the Discover sessions, January 2018.

Coded Element	Quotations about Hindrances to Physical Activity (Walking) and Access (−, Negative)
Parks/playgrounds	“A tap or drinking bubbler would be nice in the park because if you do have children or people here for a small picnic there is no way of getting hold of water” “Been walking for a little while and noticed there are no seats in the park this far I can see” “This gravel path through the park where there is a little arbour and shrubs, it’s great but there is some trimming that needs to be done so folks don’t get caught up with it”
Footpaths	“Raised paving in the footpath (is) a trip hazard” “Seems to be a loose gravel path (and) can’t quite see where it’s heading; but maybe it wouldn’t be a good (path) for people with walkers” “This pathway is too narrow for more than one person, but it may be difficult to widen it”
Traffic related safety/Parking	“I think the sign is misleading and (I am) not sure which side people would be designated to walk. An actual sign on the pathway would be beneficial” “Car park is always full; difficult for some people to be able to park in the car park” “This is the entrance from X street to the car park and the pedestrian entrance to the gymnasium (as well) everyone who goes (to the gym) has to walk up and down the hill; a zebra crossing down the hill would be useful; also traffic flow in-and-out is confusing for everyone”
